# Demonstration of immune responses against devil facial tumour disease in wild Tasmanian devils

**DOI:** 10.1098/rsbl.2016.0553

**Published:** 2016-10

**Authors:** Ruth Pye, Rodrigo Hamede, Hannah V. Siddle, Alison Caldwell, Graeme W. Knowles, Kate Swift, Alexandre Kreiss, Menna E. Jones, A. Bruce Lyons, Gregory M. Woods

**Affiliations:** 1 Menzies Institute for Medical Research, University of Tasmania, Hobart, Tasmania 7000, Australia; 2 School of Medicine, University of Tasmania, Hobart, Tasmania 7000, Australia; 3 School of Biological Sciences, University of Tasmania, Hobart, Tasmania 7001, Australia; 4 Centre for Biological Science, University of Southampton, Highfield Campus, Southampton SO17 1BJ, UK; 5 Department of Primary Industries, Parks, Water and Environment, Hobart, Tasmania 7000, Australia

**Keywords:** Tasmanian devil, devil facial tumour disease, immune response, transmissible cancer, emerging infectious disease

## Abstract

Devil facial tumour disease (DFTD) is a recently emerged fatal transmissible cancer decimating the wild population of Tasmanian devils (*Sarcophilus harrisii*). Biting transmits the cancer cells and the tumour develops in the new host as an allograft. The literature reports that immune escape mechanisms employed by DFTD inevitably result in host death. Here we present the first evidence that DFTD regression can occur and that wild devils can mount an immune response against the disease. Of the 52 devils tested, six had serum antibodies against DFTD cells and, in one case, prominent T lymphocyte infiltration in its tumour. Notably, four of the six devils with serum antibody had histories of DFTD regression. The novel demonstration of an immune response against DFTD in wild Tasmanian devils suggests that a proportion of wild devils can produce a protective immune response against naturally acquired DFTD. This has implications for tumour–host coevolution and vaccine development.

## 1. Introduction

The Tasmanian devil is the world's largest carnivorous marsupial and unique to Tasmania, the island state of Australia. The species is listed as Endangered owing to mortality from devil facial tumour disease (DFTD) [1]. The disease is a transmissible cancer, first observed in 1996 in the far northeast of the state. It is now found throughout the majority of the devil's geographical range [2]. DFTD is transmitted when susceptible and infected individuals bite each other and is considered invariably fatal, with most animals dying within 6–12 months of the tumour first appearing [3].

In 2015, a second transmissible facial cancer was reported in Tasmanian devils in the southeast of the state [4]. This second cancer was named DFT2, and in the report the original cancer was termed DFT1, with DFTD denoting both. This paper follows the nomenclature and refers to DFT1. The disease is transmitted as an allograft [5] and three explanations were initially suggested to explain the lack of immune rejection: the limited genetic diversity of the species; the unknown competency of the devil's immune system; and the immune evasion mechanisms of the tumour [2]. All research to date addressing these possibilities suggests that it is the successful immune evasion strategies employed by the tumour cells that are primarily responsible for transmission of DFT1 [2,6,7].

Current DFT1 research suggests that a major mechanism of immune escape is downregulation of the major histocompatibility complex class I molecule (MHC-I) [7]. MHC-I cell surface expression occurs on all nucleated cells and allows the immune system to recognize foreign or infected cells. Some cancers fail to express surface MHC-I, a mechanism that contributes to evasion of the host's T cell response. The only other naturally occurring transmissible cancer to affect a mammalian species is canine transmissible venereal tumour (CTVT) in domestic dogs. CTVT also adopts MHC-I downregulation in its progressive phase [8]. However, after three to four months of tumour growth, there is increased surface MHC-I expression resulting in a host alloresponse. This is demonstrated by host antibody production and T lymphocyte infiltration of the tumour resulting in tumour stabilization or regression and immunological memory. MHC-I expression is associated with the presence of inflammatory cytokines [8,9]. Likewise, DFT1s downregulation of surface MHC-I can be reversed *in vitro* by treatment of DFT1 cells with the inflammatory cytokine interferon gamma (IFN-γ) [7].

Downregulation of MHC-I provides an explanation for DFT1 transmission and is believed to be responsible for the lack of a T-cell-mediated immune response against the tumour. The long-standing assumptions are that DFT1 always escapes the devil's immune system, and that the disease is invariably fatal. We re-examined these assumptions by analysing serum and tumour samples from a population of wild devils to detect the presence of anti-DFT1 immune responses.

## 2. Material and methods

Serum samples collected from 52 devils between 2008 and 2014 from a closely monitored population in northwestern Tasmania were evaluated for the presence of IgG antibodies against DFT1 cells. This was done via indirect immunofluorescence and flow cytometry with the median fluorescence intensity (MFI) of each sample recorded [10]. For 45 of the individual devils, multiple serum samples collected over an extended period were analysed. Serum samples were tested against DFT1 cells not expressing MHC-I, referred to as MHC-I^−ve^ DFT1 cells, and separately against DFT1 cells treated with IFN-γ to induce cell surface expression of MHC-I [7], referred to here as MHC-I^+ve^ DFT1 cells. Sera from a translocated population of captive born devils living in wild conditions on a DFTD-free island were used as the negative control.

Of the 52 devils, 34 either had DFT1 at the start or developed DFT1 during the course of sampling. Where tumour biopsies were available, histopathological examination included identification of tumour-infiltrating immune cells. Immunohistochemistry using anti-MHC-II antibody to identify antigen-presenting cells and anti-CD3 antibody to identify T lymphocytes in the biopsy was performed where indicated [11]. Where immunocytochemistry (ICC) was performed on tumour fine needle aspirates (FNAs), the samples were stained for periaxin, a positive marker for DFT1 cells [12], and for β_2_ microglobulin (β_2_m), a component of the MHC-I molecule.

Detailed methods regarding serum and tumour sample collection, and analyses are provided in the electronic supplementary material.

## 3. Results

Of the 52 devils, 46 had no detectable serum IgG antibody against either MHC-I^+ve^ or MHC-I^−ve^ DFT1 cells. The remaining six devils (referred to here as TD1–6) had serum IgG antibody against MHC-I^+ve^ DFT1 cells, but not MHC-I^–ve^ cells. None of these six devils had clinical signs of DFT1 at initial sample collection, but developed DFT1 at some stage during sample collection (table 1).

**Table 1.** DFTD and antibody (Ab) status of six Tasmanian devils exhibiting anti-DFT1 responses. Serum Ab column: ‘/’, no serum sample collected; ‘negative’, same as MFI control; ‘medium’, 2–4× MFI control; and ‘high’, >4× MFI control. YOB, year of birth; FNA, fine needle aspirate.

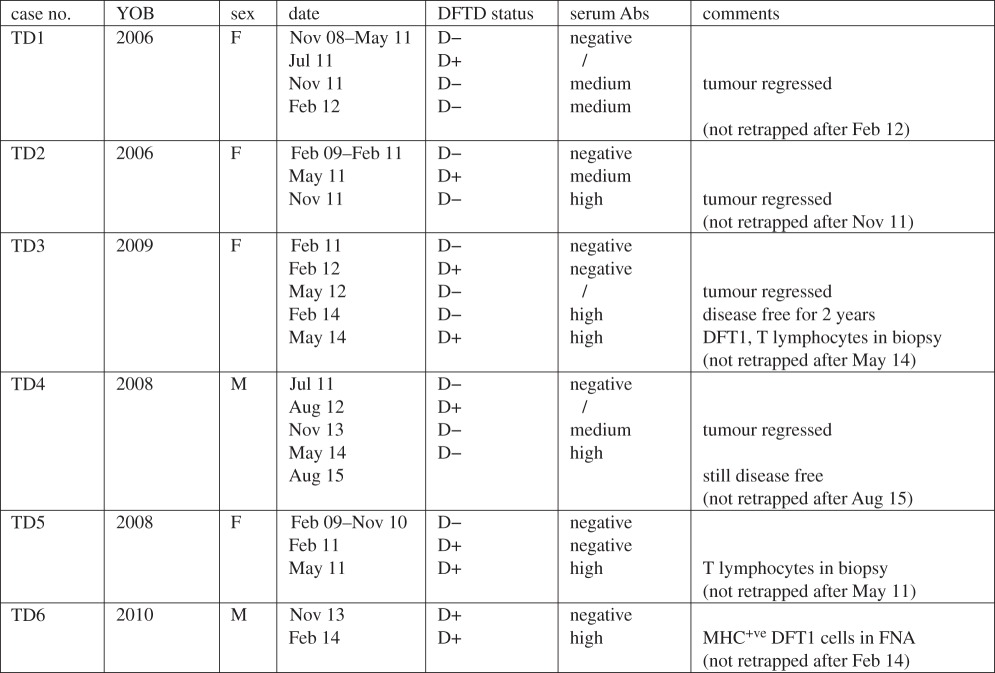

**Figure 1.**
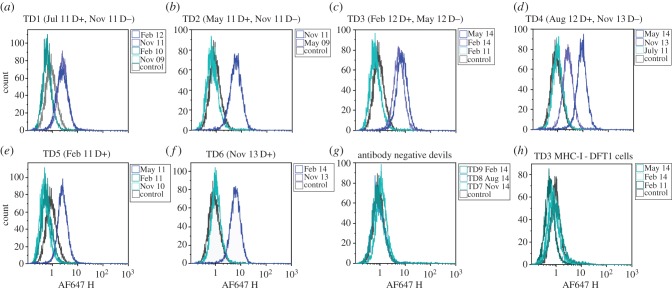
Flow cytometric analysis of anti-DFT1 antibody responses. (*a*)–(*f*) IgG serum antibody results of TD1–6 against MHC-I^+ve^ DFT1 cells compared with negative control. In brackets are the dates each devil was first observed with DFT1 (D+) and when the tumour was no longer present (D−); (*g*) representative results from three of the 46 devils that had no serum antibody; (*h*) negative results of TD3 for serum IgG against MHC-I^−ve^ DFT1 cells, representative of TD1–6.

Multiple serum samples from each of the six devils were analysed and for each devil the earliest sample had the same MFI as the negative control. After these devils showed clinical signs of DFT1, they developed anti-DFT1 antibodies (figure 1). Remarkably, DFT1 regression occurred in four of the six devils that had seroconverted (TD1, TD2, TD3 and TD4). When each devil was retrapped between four and 15 months after DFT1 was first noted, their tumours were no longer visible and anti-DFT1 antibodies were detected. TD1 and TD2 were not retrapped after the regression was observed. TD3 remained disease free for 2 years following tumour regression but at the age of 5 years, a tumour biopsy confirmed recurrence of DFT1. Serum antibodies persisted in TD3 at this time and tumour-infiltrating MHC-II positive cells and CD3 positive T lymphocytes were present in the biopsy. TD4 remained disease free for 3 years to the age of 6, beyond which it was not retrapped (6 years is considered the maximum lifespan for a wild devil).

**Figure 2.**
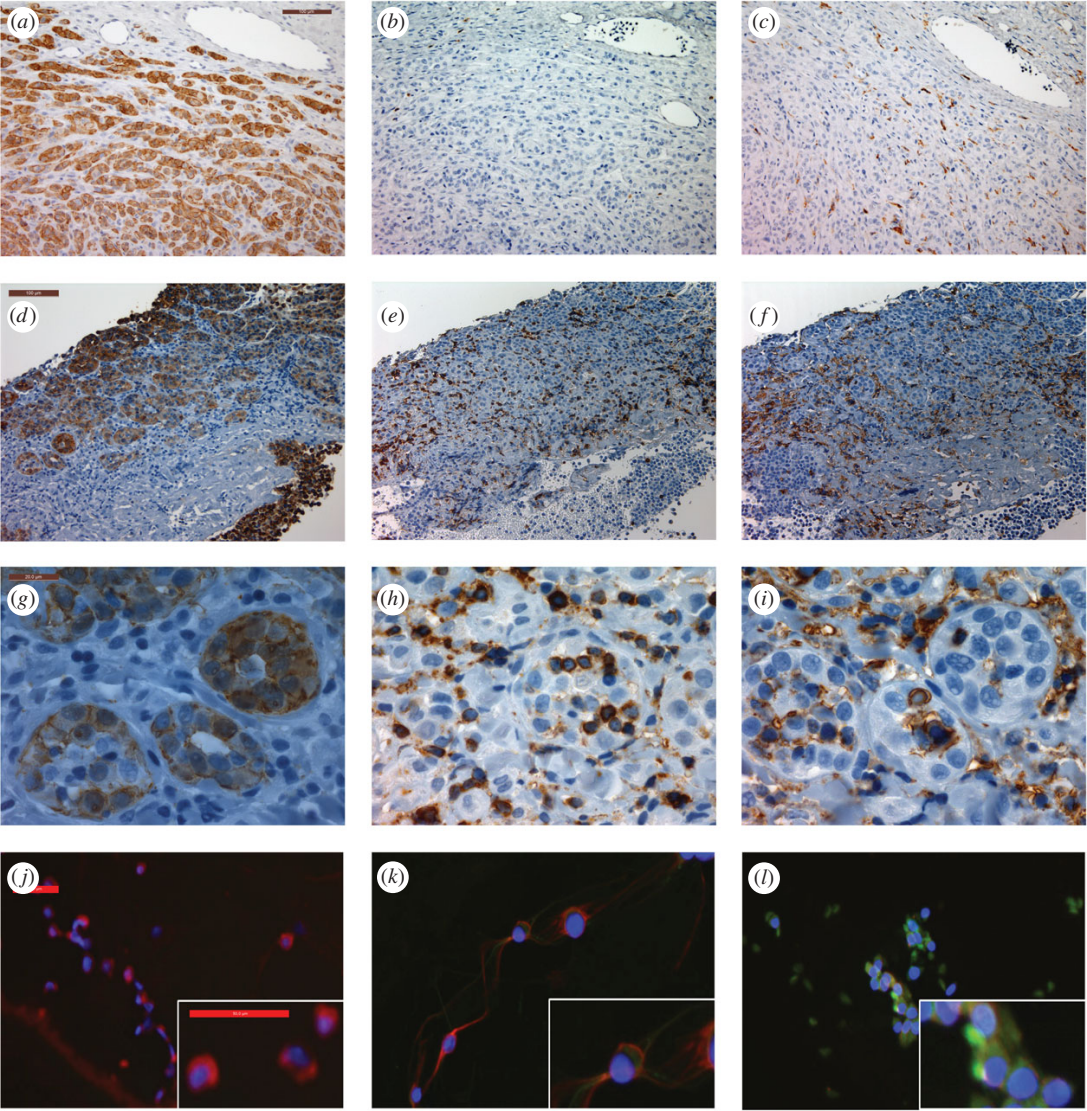
Evidence for immune cell infiltration of DFT1 or MHC-I expression of DFT1 cells. (*a*–*i*) IHC staining of DFT1 tumour biopsies. Top and middle rows taken at 20× magnification, scale bar indicates 100 µm. Bottom row taken at 100× magnification, scale bar indicates 20 µm. Positive cells for each marker are brown; haematoxylin (blue) is the counter stain. (*a*,*d*,*g*) periaxin, marker for DFT1 cells; (*b*,*e*,*h*) CD3, marker for T lymphocytes; (*c*,*f*,*i*) MHC-II, marker for antigen-presenting cells; (*a*–*c*) typical DFT1 biopsy with no evidence of immune response; (*d*–*f*) tumour biopsy from TD5 showing infiltration of CD3 and MHC-II positive cells throughout the tumour; (*g*–*i*) tumour biopsy from TD5 showing immune cells infiltrating DFT1 cell clusters. (*j*–*l*) ICC of DFT1 cells with periaxin (red), and β_2_m (green) to identify MHC-I surface expression: (*j*) DFT1 cells from culture; (*k*) DFT1 FNA from TD6 collected in November 2013; (*l*) DFT1 FNA from TD6 collected in February 2014. (ICC images taken at 20× magnification, each scale bar indicates 50 µm.)

Tumour regression for TD5 and TD6 was not observed; however, their tumour samples showed interesting features. At the time of TD5's seroconversion, the biopsy had tumour infiltration of MHC-II and CD3 positive cells (figure 2). TD6 had tumour FNAs and serum collected when first trapped and again three months later. Cells from the initial FNA were periaxin positive and faintly positive for β_2_m (figure 2*j*,*k*); however, three months later, coinciding with seroconversion, the periaxin positive cells were strongly positive for β_2_m, indicating MHC-I expression by the DFT1 cells (figure 2*l*). Neither TD5 nor TD6 was retrapped following seroconversion. In contrast with TD3, TD5 and TD6, the tumour samples from devils without serum antibody did not show significant tumour infiltration of immune cells or β_2_m staining.

## 4. Discussion

The immune escape mechanisms of DFT1 play a significant role in its successful transmission and tumour development. While anti-DFT1 immune responses have been induced in captive devils by immunizing with killed DFT1 cell preparations [10], no convincing evidence for immune responses against DFT1 have previously been identified in wild devils. Here we report the first evidence, we believe, of anti-DFT1 immune responses occurring in wild Tasmanian devils exposed to DFT1.

The serum antibodies directed against IFN-γ treated DFT1 cells (MHC-I^+ve^) found in six devils may have resulted from an initial immune response against the primary tumour and subsequent IFN-γ release. This may have upregulated MHC-I expression on the DFT1 cells, resulting in an immune response against these modified cells. Our results indicate that this response is initiated by infiltrating T lymphocytes, which, although rare, have been documented in at least one case of DFT1 and associated with tumour cell surface expression of MHC-I [7]. What caused the initial immune response is not clear. However, the increase in MHC-I expression on DFT1 cells potentially provided a mechanism for T-cell-mediated killing of tumour cells and ultimately tumour stabilization or regression. Antibody production, in the form of IgG, provides confirmatory evidence that an anti-DFT1 immune response had been generated. It was not possible to accurately measure IgM levels via flow cytometry to determine if IgM/IgG ratios predict protection, as suggested by Ujvari *et al.* [13]. The IgG antibodies could facilitate tumour cell killing via antibody-dependent cell-mediated cytotoxicity. Although there are significant differences between CTVT and DFT1, they share characteristics of transmissibility and MHC-I downregulation. Indeed the development of IgG antibodies against DFT1 cells may parallel what is believed to occur in CTVT cases: after the canine tumour has established there are increased numbers of MHC-I^+ve^ CTVT cells discernible by immunohistochemistry and immunocytochemistry, and the development of serum IgG antibodies against CTVT cells occurs [8,9]. The experimentally induced CTVTs tend to regress [8], whereas the naturally occurring tumours seem to remain in equilibrium as locally invasive tumours with metastases being uncommon [14]. It is probable that this equilibrium or regression occurs as a result of the increased MHC-I expression of the tumour cells. The consecutive tumour FNAs taken from TD6 showed increased intensity of β_2_m surface staining indicative of increased MHC-I expression on the DFT1 cells. Upregulation of MHC-I, along with seroconversion occurring at that time, indicates that DFT1 and CTVT may share additional characteristics of disease progression.

While there has been no observed reduction in the demographic effect of DFT1 in the local population of this study, this evidence indicates that DFT1 does not always escape detection by the immune system, and death may not be the inevitable outcome of infection. The naturally occurring immune responses against DFT1 may enable identification of significant tumour antigens and thus advance DFT1 vaccine development. If there is a heritable component to the immune response, over time selection should favour those individuals that are able to recognize the tumour, with increased survival and, therefore, lifetime reproductive output leading to increased representation of these devil lineages and increased survival of wild populations.

In summary, we have demonstrated a naturally occurring immune response against DFT1 in this population of wild devils. Our findings highlight the value of monitoring disease at the individual level where ongoing microevolutionary changes can be detected and permit evaluation of their impact on the disease trajectory and epidemic outcome at a population level.

## Supplementary Material

Supplementary material from “Demonstration of immune responses against devil facial tumour disease in wild Tasmanian devils”

## Data accessibility

The datasets supporting this article have been uploaded as part of the electronic supplementary material. All manuscript figures belong to the authors.
