# Supplementary material for: Demonstration of immune responses against devil facial tumour disease in wild Tasmanian devils
Source: Biol Lett. 2016 Oct;12(10):20160553. doi: 10.1098/rsbl.2016.0553 (PMC5095191; doi:10.1098/rsbl.2016.0553)
Supplement: Supplementary material from “Demonstration of immune responses against devil facial tumour disease in wild Tasmanian devils” [file rsbl20160553supp1.docx]

**Demonstration of immune responses against devil facial tumour disease in wild Tasmanian devils**

Ruth Pye ^1^, Rodrigo Hamede ^2^, Hannah Siddle ^3^, Alison Caldwell ^3^, Graeme Knowles ^4^, Kate Swift ^4^, Alexandre Kreiss ^1^, Menna Jones ^2^, A. Bruce Lyons ^5^, Greg Woods ^1^

1. Menzies Institute for Medical Research, University of Tasmania, Hobart, TAS 7000, Australia

2. School of Biological Sciences, University of Tasmania, Hobart, TAS 7001, Australia

3. Centre for Biological Science, University of Southampton, Highfield Campus, Southampton SO17 1BJ, United Kingdom

4. DPIPWE Tasmania AHL, Launceston TAS 7250

5. School of Medicine, University of Tasmania, Hobart, TAS 7000, Australia

**Supplementary information**

**Materials and Methods**

**Individual devils:**

Blood samples were collected from a total of 52 devils, ages ranging from 1 to 6 years, with 34 females and 18 males (Table S1). Tumour samples were collected from a total of 20 devils. Tumour sample collection was dependent on size and location of the tumour, and was not always possible in a field setting. Tumour size was measured by recording length, width and depth with 15cm caliMax calipers.

A diagnosis of DFT1 was made based on clinical findings supported by histopathological examination of tumour biopsies for 10 devils. In cases where histopathological samples were not taken, fine needle aspirate (FNA) collection and either cytological examination (1 devil), cell culture and karyotype (6 devils), or immunocytochemistry (ICC) (5 devils, 2 of which also had biopsies collected) were taken from cutaneous or oral raised nodular or ulcerated lesions. For the remaining 14 devils, there was visual assessment of tumour location and appearance according to previously described classification methods(1) (Table S3).

There were 6 devils (TD1 to TD6) that had serum antibody. These 6 devils each had DFT1 at some stage during sample collection: DFT1 assessment was made in TD1, TD2 and TD3 by visual examination; TD4 by cytology of the FNA; TD5 by histopathology; TD6 by ICC of the FNAs.

Blood was collected from a further 20 devils from a DFTD free island to use as the negative control serum (Table S2). The serum samples had been collected >6 months after release of the devils on to the island. The adult (> 1 year old) females were noted to have slightly higher MFI’s than the other cohorts so their sera were used as the negative control for the wild devils from West Pencil Pine.

**Table S1. Summary of serum samples collected and analysed from Tasmanian devils from West Pencil Pines**

| **Age (years)** | **1** | **2** | **3** | **4** | **5** | **6** | **Total** |
| --- | --- | --- | --- | --- | --- | --- | --- |
| Number of devils | 16 | 15 | 11 | 4 | 4 | 2 | 52 |
| DFT1+ (including those with regressed tumours) | 5 | 10 | 11 | 3 | 3 | 2 | 34 |
| DFT1- | 11 | 5 | 0 | 1 | 1 | 0 | 18 |
| Females | 8 | 8 | 10 | 3 | 4 | 1 | 34 |
| Males | 8 | 7 | 1 | 1 | 0 | 1 | 18 |
| Females DFT1+ | 2 | 6 | 10 | 2 | 3 | 1 | 24 |
| Males DFT1+ | 3 | 4 | 1 | 1 | 0 | 1 | 10 |
| Number of devils that had only 1 serum sample analysed | 5 | 2 | 0 | 0 | 0 | 0 | 7 |
| Number of devils that had multiple serum samples analysed | 11 | 13 | 11 | 4 | 4 | 2 | 45 |
| Number of devils that had serum taken prior and subsequent to becoming DFT1+ (including those with regressed tumours) | 0 | 4 | 7 | 2 | 2 | 2 | 17 |
| Number of devils that had their final serum sample taken at time of first clinical signs | 3 | 6 | 4 | 1 | 0 | 0 | 14 |
| Number of DFT1- devils that had multiple serum samples analysed | 8 | 4 | 0 | 1 | 1 | 0 | 14 |

**Table S2. Summary of samples collected and analysed from Tasmanian devils from Maria Island (DFTD free island) used as the negative control serum.**

|  | **1 year old** | **Adult (> 1 year old)** |
| --- | --- | --- |
| **Males** | 5 | 5 |
| **Females** | 5 | 5 |

**Serum sample collection:**

Between 2 and 5 ml blood was collected from the jugular vein with a 21G hypodermic needle and a 5 ml syringe (University of Tasmanian Animal Ethics Approval (A0013326), and transferred directly into clot activating tubes. Samples were centrifuged at 1000G for 10 minutes and the serum pipetted off and stored frozen at -20C° or -80C°.

**Tumour biopsy collection:**

Samples were collected using 4mm biopsy punches (Kai) and fixed in 10% neutral buffered formalin.

**Tumour fine needle aspirate collection for cytological examination or immunocytochemistry:**

Tumour aspirates were collected with a 21G or 23G hypodermic needle and 5ml syringe and expressed on to glass microscope slides, air dried and stained with May Grunwald-Giemsa stain. Aspirates collected for immunocytochemistry analysis were placed directly into 1ml vials of 4% paraformaldehyde, and after approximately 10 days (on return from the field) were centrifuged and resuspended in phosphate buffered saline for long term storage.

**Tumour fine needle aspirate collection for cell culture and karyotype:**

Tumour aspirates were collected as described above and placed directly into 5ml of transport medium (RPMI 1640 medium with L-glutamine (Sigma-Aldrich), plus 0.1 mL of penicillin-streptomycin solution (Sigma-Aldrich,) and 5 μg amphotericin B (Sigma-Aldrich)). Within 48 hours, the cells were centrifuged at 1000rpm and transferred to 25cm culture flasks containing 10mL RPMI 1640 with L-glutamine and 10% foetal calf serum (Sigma-Aldrich). After 24-72 hours 0.1mL of demecolcine at 10µg/mL (Sigma-Aldrich) was added to each culture and incubated for 4 hours. The cells were centrifuged for 10 minutes at 1000 rpm. Supernatant was discarded and the cell pellet was slowly resuspended in 7mL of hypotonic 0.075M KCl and placed in a water bath at 37°C for 18 minutes. 2mL of chilled Carnoy’s fixative (3:1 ratio of methanol to acetic acid) was added and the tubes centrifuged for 10mins at 1000 rpm. After removing the supernatant the pellet was gently resuspended in Carnoy’s fixative and stored at 4°C overnight. The following day the cells were spun and resuspended in 3 changes of fresh fixative. The cells were then resuspended in enough fixative to form a milky suspension. This suspension was dropped onto clean, frozen microscope slides from 10cm to ensure chromosome spread. Slides were allowed to dry and then placed in an oven at 57°C for 3 days before banding. G-banding was conducted by treating slides with a 0.15% solution of trypsin for up to 30 seconds before staining with Leishmann’s stain for 2½minutes, then mounting with Leica mounting medium (Leica Microsystems) for analysis.

G-banding analysis was performed using a Leica DM 2000 microscope (Leica Microsystems) and photographed with a Leica DFC 420 C camera (Leica Microsystems). Karyotypes were made using VideoTest Karyo 3.1 (VideoTest). 50 Metaphases were analysed for each sample.

**Cell culture and serum sample analysis:**

Cell culture and flow cytometry methods are similar to those described in (2). Three different DFT1 cell lines (C5065 = strain 3, year of origin 2007; 1426 = strain 2, year of origin 2005; Ed = strain 2, year of origin 2012) were maintained in T75 flasks with complete RPMI medium, consisting of RPMI 1640 medium (Life Technologies, Grand Island, USA) supplemented with 10% vol/vol foetal calf serum (Bovogen Biological, Victoria, Australia), 5mM L-glutamine (Sigma-Aldrich, St Louis, USA) and 100IU gentamicin sulphate (Pfizer, Western Australia, Australia) at 35°C in a humidified atmosphere of 5% CO_2_ in air.

DFT1 cells were treated with recombinant devil INF-γ (50ηg/ml, 24 hours incubation) to induce cell surface expression of MHC-I, confirmed by flow cytometry with positive staining for Beta 2 microglobulin (β_2_m), a component of the MHC molecule (3). These cells are referred to here as MHC-I ^+ve^ DFT1 cells. Untreated cells are negative for β_2_m staining and are referred to here as MHC-I ^–ve^ DFT1 cells.

DFT1 cells were washed 3 times with FACS buffer (PBS + 1% BSA + 0.1% NaN3) before each of the following steps. Serum samples were diluted 1:50 with FACS buffer and incubated with approximately 2 x 10^5^ MHC-I ^+ve^ DFT1 cells/ well and separately with 2 x 10^5^ MHC-I ^–ve^ DFT1 cells/ well in 96 well plates for 1 hour on ice. Cells were then incubated with a monoclonal mouse anti-devil IgG antibody for 30 minutes on ice. Finally, cells were incubated with a fluorochrome labeled goat anti-mouse IgG antibody (Alexa Fluor® 647) for 30 minutes on ice. They were re-suspended in 200μl FACS buffer containing 3μM propidium iodide (Sigma-Aldrich, St Louis, USA) to allow gating of dead cells, and analysed by flow cytometry (BD Canto II). Cells labeled with secondary and tertiary antibodies only gave no background fluorescence. The mouse IgG2b isotype (Bioscience) controls showed the same MFI as the no-serum controls. The same (positive/ negative) antibody results were achieved when the sera was tested against the 3 different lines. Antibody responses were classified as “negative” < 1.5 x MFI of negative control; “low” 1.5 – 2 x MFI of negative control; “medium” 2 – 4 x MFI of negative control; or “high” > 4 x MFI of negative control.

**Table S3 Summary of techniques used to identify DFT1 in the 34 DFT1+ devils**

| **Diagnostic technique** | **No. of DFT1 cases** | **Veterinary pathologist report/ periaxin positive** | **Devils with serum antibody i.e. TD1 – TD6** |
| --- | --- | --- | --- |
| Visual diagnosis | 14 | / | TD1, TD2, TD3 |
| Histopathology (biopsy) | 11 (including TD3’s DFT1 recurrence) | 11 | TD5  (TD3 when DFT1 recurred in 2014) |
| Cytology: May Grunwald-Giemsa stain | 1 | 1 | TD4 |
| Cytology: immunocytochemistry | 5 (2 of these also had biopsies from which a histology diagnosis was made) | 5 | TD6 |
| Cell culture & karyotype | 6 | 6 | / |

**Immunohistochemistry:**

Methods are the same as those described in (4) and (5). DFT1 tumour biopsies were fixed in 10% buffered formalin, embedded in paraffin wax and sectioned (3 μm) onto 3-aminotriethoxysilane (Sigma-Aldrich) coated slides. Standard hematoxylin and eosin (H&E) staining was performed at the Animal Health Laboratory, Tasmania, or at the Anatomical Pathology Department of the Royal Hobart Hospital.

For immunohistochemistry, sections were initially deparaffinized in a histology oven at 60 C for 15 minutes followed by two 5-minute washes in xylene and then rehydrated through successive graded ethanol solutions and washed for 5 minutes in distilled water. Heat-induced antigen retrieval was performed in citrate buffer, pH 6 (Dako, Carpinteria, CA) using an electric pressure cooker at medium heat for 10 minutes, followed by a 20-minute cooling period at room temperature.

The slides were preincubated with serum-free blocking solution (Dako) for 30 minutes and then incubated with the primary antibody (rabbit anti-human CD3, Dako A0452, mouse anti-human HLA-DR (MHC II+ cells) Dako M0746, or rabbit antihuman periaxin, Sigma/HPA 001868) for 1 hour. Endogenous peroxidase activity was then quenched by treating the slides in 3% hydrogen peroxide in phosphate-buffered saline (PBS) for 10 minutes at room temperature. Signal detection was carried out using the anti-rabbit (K4003) Dako EnVision System, HRP, (Dako). Liquid DAB1 Substrate Chromogen System (Dako) was then added to each slide and incubated for 10 min. The sections were counterstained with hematoxylin and mounted.

**Histopathology and immunohistochemistry interpretation:**

Histopathology was assessed by a veterinary pathologist (GK). Confirmation of DFT1 by H&E stained sections was based on histopathologic findings consistent with Loh et al (6). Assessment of tumour (intratumoural) infiltrating lymphocytes in anti-CD3 antibody stained sections was based on the method outlined by Zhang et al (7) and reviewed by Gooden et al (8). A similar method for counting the number of intratumoural infiltrating positive MHCII cells was performed.

The positive control tissue for anti-CD3 and anti-MHC-II antibodies was lymph node tissue. CD3 positive control demonstrated cytoplasmic and cell membrane staining of parafollicular lymphocytes within the cortex of the lymph node. MHC-II positive control demonstrated staining of B lymphocytes cell wall and cytoplasm within the germinal centres in the lymph node cortex and also scattered macrophages within cortical parafollicular sinuses and medullary sinuses in the lymph node.

**Table S4 Table showing numbers of positive staining cells per high power field (HPF) of 9 DFT1 biopsies stained with anti-CD3 antibody and (in 2 cases) anti-MHC-II antibody infiltrating the tumour. Note TD3 and TD5 had serum antibody against DFT1 cells. The remaining devils had no serum antibody recorded.**

10 high powered fields counted for all tumours except for TD3 (7 x HPF) and TD5 (3 x HPF) because these were the only areas for the tumours.

| **Animal ID** | **CD3+ tumour infiltrating T cells/ HPF ± SD** | **MHCII+ tumour infiltrating cells/ HPF ± SD** |
| --- | --- | --- |
| TD 416 | 0 | / |
| TD417 | 1± 1 | / |
| TD421 | 0 | / |
| TD433 | 0 | / |
| TD439 | 0 | / |
| TD455 | 0 | / |
| TD473 | 0 | / |
| TD3 | 4±2 | 4± 2 |
| TD 5 | 20± 2 | 18±2 |

**Immunocytochemistry:**

100 μl of each FNA sample were seeded onto poly-L-lysine (Sigma P4707) coated slides. Slides were incubated for 60 min at room temperature. Excess cell suspension was aspirated and slides placed in PBS for 10 min. Cells were incubated in ice-cold methanol for 10 min and air-dried, before incubation in blocking buffer (10% goat sera in PBS) for 30 min to minimize any non-specific absorption of antibodies. Cells were incubated in anti-devil β_2_m antibody (3) at 20 μg/ml and periaxin (Sigma HPA001868) at 1:300 in blocking buffer for 30 min. Cells were washed twice in PBS for 10 min and then 5 min. The secondary antibodies (goat anti-mouse Alexa Fluor® 488 and goat anti-rabbit Alexa Fluor® 647) were diluted 1:1000 in PBS and cells were incubated for 30 min. Cells were washed three times in PBS for 5 min. Slides were mounted with DAPI mounting media (Fluoroshield™ with DAPI, Sigma).

**REFERENCES**:

1. Hawkins CE, Baars C, Hesterman H, Hocking GJ, Jones ME, Lazenby B, et al. Emerging disease and population decline of an island endemic, the Tasmanian devil Sarcophilus harrisii. Biol Conserv. 2006;131(2):307-24.

2. Kreiss A, Brown GK, Tovar C, Lyons AB, Woods GM. Evidence for induction of humoral and cytotoxic immune responses against devil facial tumor disease cells in Tasmanian devils (Sarcophilus harrisii) immunized with killed cell preparations. Vaccine. 2015.

3. Siddle HV, Kreiss A, Tovar C, Yuen CK, Cheng Y, Belov K, et al. Reversible epigenetic down-regulation of MHC molecules by devil facial tumour disease illustrates immune escape by a contagious cancer. Proc Natl Acad Sci U S A. 2013;110(13):5103-8.

4. Tovar C, Obendorf D, Murchison EP, Papenfuss AT, Kreiss A, Woods GM. Tumor-specific diagnostic marker for transmissible facial tumors of Tasmanian devils: immunohistochemistry studies. Vet Pathol. 2011;48(6):1195-203.

5. Kreiss A, Obendorf DL, Hemsley S, Canfield PJ, Woods GM. A histological and immunohistochemical analysis of lymphoid tissues of the Tasmanian devil. Anat Rec (Hoboken). 2009;292(5):611-20.

6. Loh R, Bergfeld J, Hayes D, O'Hara A, Pyecroft S, Raidal S, et al. The pathology of devil facial tumor disease (DFTD) in Tasmanian Devils (Sarcophilus harrisii). Vet Pathol. 2006;43(6):890-5.

7. Zhang L, Conejo-Garcia JR, Katsaros D, Gimotty PA, Massobrio M, Regnani G, et al. Intratumoral T cells, recurrence, and survival in epithelial ovarian cancer. N Engl J Med. 2003;348(3):203-13.

8. Gooden MJ, de Bock GH, Leffers N, Daemen T, Nijman HW. The prognostic influence of tumour-infiltrating lymphocytes in cancer: a systematic review with meta-analysis. Br J Cancer. 2011;105(1):93-103.
